# Interactome of FMRP-N-tat therapeutic unveils key interactions for cellular function in Fragile X neurons

**DOI:** 10.1016/j.jbc.2025.110341

**Published:** 2025-06-04

**Authors:** Kévin Leguay, Mariana Acevedo, Eva Colic, Preya U. Patel, Saeideh Shamsi, Helen LB. Chan, Sharon Sun, Daneck Lang-Ouellette, Benny Chan, Xiaoqin Zhan, Ray W. Turner, Joseph Mancini, Oliver A. Kent

**Affiliations:** 1 Department of Pharmacology, adMare BioInnovations, Montréal, Quebec, Canada; 2 Department of Pharmacology, adMare BioInnovations, Vancouver, British Columbia, Canada; 3 Pharmaceutical Development Group, adMare BioInnovations, Vancouver, British Columbia, Canada; 4 Hotchkiss Brain Institute, University of Calgary, Calgary, Alberta, Canada

**Keywords:** FMRP, Fragile X syndrome, cell-penetrating peptide, peptide interaction, mass spectrometry

## Abstract

Therapeutic protein replacement has demonstrated preclinical and clinical efficacy in neurological disorders but has not been used clinically for Fragile X syndrome (FXS), a genetic neurodevelopmental disorder caused by loss of Fragile X messenger ribonucleoprotein (FMRP). FXS results from a triplet repeat expansion of more than 200 CGG repeats in the 5′-UTR of the *FMR1* gene leading to epigenetic silencing of FMRP. Currently, no clinically approved disease-modifying treatments for FXS exist. Recently, a tat-conjugated FMRP fragment encompassing residues 1 to 297 (FMRP N-tat) was shown to restore aspects of neuronal function in a mouse model of FXS. Promising *in vivo* data hinted to the therapeutic potential of FMRP N-tat. Herein, affinity purification mass spectrometry was used to identify the FMRP N-tat interactome in tsA-201 *FMR1* knockout cells and FXS patient iPSC-derived neurons. The FMRP N-tat interactome included RNA binding proteins and constituents of the ribosome, which aligned closely with the known functions of FMRP. Further, the FMRP N-tat associated proteins included FXR2, STAU1, TRIM28, C1QBP, VDAC2, and several ribosomal proteins to regulate mRNA stability, cellular stress responses, mitochondrial function, and translation. The results highlight the potential of FMRP N-tat to orchestrate assembly of factors to correct lost function in FMRP deficient cells.

Protein-based therapeutics are imminently becoming new paradigms in disease treatment encompassing multiple classes and functions of proteins (1, 2). In addition to viral approaches for gene replacement, protein replacement therapy includes the use of exogenously produced proteins to replace missing endogenous proteins. The use of therapeutic proteins has garnered the attention of pharmaceutical research, as these molecules display desirable attributes including high selectivity and good tolerability (2). Indeed, protein therapy is emerging as a tractable strategy for neurological diseases such as Alzheimer’s disease, Parkinson’s disease, and Huntington’s disease; however, protein therapy has not yet been utilized clinically for FXS (3, 4, 5, 6, 7).

FXS is a heritable intellectual and autism spectrum disorder (ASD) resulting from loss of Fragile X messenger ribonucleoprotein (FMRP) found on the X chromosome (8). FXS afflicts 1 in 4000 males and 1 in 7000 females and is the most common monogenic cause of ASD (9). FXS is a triplet repeat disorder caused by expansion of the approximately 30 CGG triplet repeats normally observed in the 5′-UTR of the *FMR1* gene to more than 200 CGG repeats (10, 11). Expansion leads to DNA hypermethylation, transcriptional inactivation, and silencing of FMRP protein expression. Since FMRP functions to regulate translational control in neurons, dysregulated translation resulting from loss of FMRP is thought to underlie the pleiotropic molecular and clinical manifestations of FXS (12). Indeed, phenotypes observed in patients, as well as those observed *in vivo* and *in vitro* models, can be attributed to dysregulated translational control and deficient protein synthesis of numerous FMRP regulated target genes (13, 14, 15, 16).

The FMRP protein is a large RNA binding protein consisting of 632 amino acids. FMRP contains three canonical RNA binding K-homology domains (KH) including a recently discovered motif KH0, at residues 126 to 206, and two centrally located KH domains called KH1 and KH2 spanning residues 206 to 422 (17, 18, 19). The N-terminal region containing the first 134 amino acids, also named NDF for N-terminal domain of FMRP, forms a stable protein that contains two tandem Agenet (also known as Tudor) domains that interact with RNA, chromatin, and other proteins (20). The NDF forms stable dimers and weak interactions with the full-length protein directed by the Agenet domains and intramolecular interactions with KH0 (20). FMRP can translocate between the nucleus and cytoplasm *via* its nuclear localization sequence (NLS) and nuclear export sequence (NES), respectively (21). However, at steady state, FMRP is found predominantly in the cytoplasm (21). The C-terminus of FMRP has arginine-glycine-glycine (RGG) motif or RGG box, involved in nonspecific RNA recognition (18). Further, the C-terminal region of FMRP has been shown to regulate its ability to phase separate into liquid droplets, likely *via* the intrinsically disordered nature of this region which may influence translational regulation (22, 23).

Early studies have shown that a short peptide fragment derived from HIV-tat (trans-activator of transcription, amino acids 48–57) acts as a cell-penetrating peptide to enable passage of protein cargo across cellular membranes with no apparent associated toxicity (24, 25). Previously, it was demonstrated that a tat-conjugated fragment of FMRP (amino acids 1–297) could restore ion channel and synaptic function in a murine model of FXS (26). The tat-conjugated FMRP 1 to 297 protein (herein referred to as FMRP N-tat) encompasses the entire NDF up to and including a fragment of the KH2 domain (Fig. 1*A*). When FMRP N-tat was introduced to *Fmr1* knockout mice by tail vein injection, it rapidly permeated the blood–brain barrier (BBB) and was detected in cortical, hippocampal, and cerebellar cells (26). The delivered FMRP N-tat restored aspects of neuronal function including mossy fiber LTP, reduced hyperactivity in adult animals, and rescued disrupted translation of select proteins associated with FXS (26). Therefore, protein replacement may be one way to effectively restore FMRP-related circuit function in FXS. Promising *in vivo* efficacy of protein replacement highlights the potential of FMRP N-tat as a therapeutic strategy for FXS.

**Figure 1:**
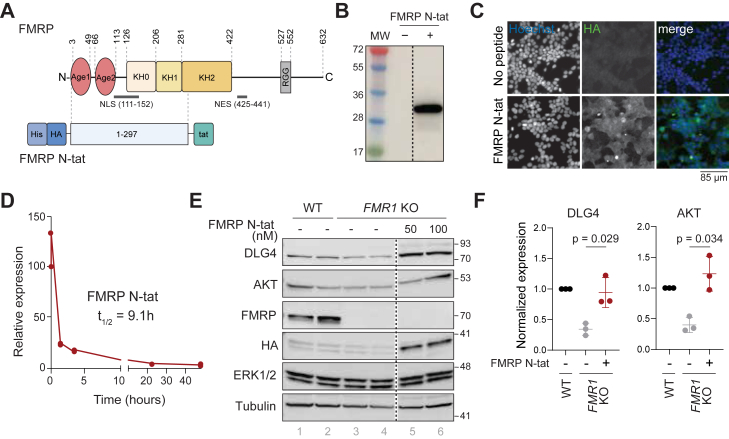
**FMRP N-tat displays biological activity in tsA-201 *FMR1* KO cells**. *A*, schematic representation of human FMRP protein domain structure based on boundaries described by Prieto *et al*. (19), and aligned to FMRP N-tat. *B*, immunoblot of FMRP N-tat recombinant protein purified from Sf9 lysates. No protein (−) and FMRP N-tat (+) resolved by SDS-PAGE/western detected by using an HA-tag antibody. The *dotted line* indicates intervening lanes that were removed from the blot. *C*, Immunolabel of FMRP N-tat uptake in HEK293 cells when applied in the culture medium at 50 nM for 18 h and detected using an anti-HA antibody (see also Fig. S1*D*). The *blue signal* corresponds to a nuclear stain (Hoesche). Images for the *green* and *blue* channels are shown in *gray* scale for clarity. *D*, Measurement of FMRP N-tat half-life in HEK293 cells after addition to the culture medium at 50 nM and processed by analysis of band intensity detected in Western blot at the indicated times. A split x-axis has been applied to highlight the early data points. Data represented as the relative expression of FMRP N-tat normalized to vinculin quantified from western blots (see Fig. S1*F*). *E*, immunoblot of tsA-201 WT and tsA-201 *FMR1* KO cells treated with FMRP N-tat (N-tat, 50 nM and 100 nM) processed 18 h after application. Immunoblots are representative of three independent experiments. FMRP marks the endogenous full-length protein and FMRP N-tat is identified by an antibody to the HA tag. ERK1/2 and Tubulin served as a loading controls. The *dotted line* indicates intervening lanes that were removed from the blot. The complete, uncropped blots are shown in Figure S1*G*. *F*, quantification of DLG4 and AKT expression in tsA-201 WT and tsA-201 *FMR1* KO cells after FMRP N-tat treatment (100 nM-treated samples) and normalized to tubulin. Each *dot* represents one independent experiment. All experiments are normalized to protein levels detected in tsA-201 WT cells. *p*-values were calculated using a one-tailed paired *t* test.

A therapeutic protein for FXS must engage the appropriate cellular machinery in FMRP-deficient cells to restore disrupted molecular functions and rescue disease-related phenotypes. In the present study, a combination of affinity purification mass spectrometry (AP-MS) coupled with crosslinking of FMRP N-tat was performed to unveil the FMRP N-tat interactome in tsA-201 *FMR1* knockout (KO) cells and iPSC-derived neurons of patient with FXS. Analysis of FMRP N-tat interactions in tsA-201 *FMR1* knockout (KO) cells and FXS patient-derived neurons revealed the ability of FMRP N-tat to restore lost molecular networks, rescuing key phenotypes including dysregulated protein synthesis and impaired mitochondrial membrane function.

## Experimental procedures

### Cell culture

HEK293 and tsA-201 cells (WT and *FMR1* KO) were cultured as previously described (27). Briefly, cells were cultured in DMEM with 10% fetal bovine serum (FBS) at 37 °C with 5% CO_2_. For iPSC work, all protocols, media, and reagents were from STEMCELL Technologies (STEMCELL.com). WC005i-FX11 to 7 iPSCs (referred to as FXS) derived from a 7-years male donor and a male *FMR1* genetically wild-type iPSC line WA01(H1) (referred to as WT) were obtained from WiCell. iPSCs were maintained in mTeSR Plus on Matrigel-coated plates. Neural progenitor cells (NPCs) were derived from single-cell suspension of iPSCs seeded into AggreWell800 24-well plates for embryoid body (EB) protocol. EBs were maintained in STEMdiff Neural induction medium. Neural rosettes were selected using STEMdiff Neural Rosette Selection Reagent and maintained in STEMdiff Neural induction medium. The resulting NPCs (day 19) were maintained in STEMdiff Neural Progenitor Medium. Patient FXS neurons were generated from NPCs in midbrain neuron differentiation medium. Cells were split into 10 cm dishes coated with poly-L-ornithine and laminin and permitted them to grow for at least 2 weeks in neuron maturation medium. For FMRP N-tat treatment, 50 nM recombinant protein was bath applied to neurons.

### Peptide cloning and purification

The insect codon optimized FMRP N-tat DNA sequence was cloned into a pFastBac vector (Fig. S1*A*) and transformed into DH10Bac-competent cells to permit expression and purification from insect cells following the Bac-to-Bac Baculovirus Expression System protocol (ThermoFisher). Insect cell culture was performed in Sf900 II media with 5% FBS. Recombinant FMRP N-tat was isolated from Sf9 cell pellets using 10 ml freshly prepared cold lysis buffer (50 mM HEPES, pH 8, 500 mM KCl, 250 mM Glucose, 5 mM MgCl_2_, 5 mM ATP, 100 mM Arginine, 1 mM DTT, 5 mM Imidazole) containing cOmplete Protease inhibitors (Roche). DTT (1 mM) was added fresh prior to lysis. The lysates were sonicated on ice for 12 cycles with 10 s on, 30 s off at 15% amplitude. The suspension was centrifuged at 37,000×*g*, 4 °C for 1-h to remove debris and membrane-bound proteins. The supernatant was loaded onto a His-Trap 1 ml column using the AKTA Express. The column was washed with 25 column volumes (CV) of wash buffer (50 mM HEPES, pH 8, 500 mM KCl, 250 mM Glucose, 5 mM MgCl_2_, 5 mM ATP, 100 mM Arginine, 1 mM DTT) with up to 10 mM of imidazole using an increasing gradient. The protein was eluted in 2-ml fractions with 15 CV of elution buffer (identical to wash buffer) with up to 200 mM imidazole gradient. Elution was performed at room temperature (RT) and eluted samples placed on ice. FMRP N-tat fractions were pooled and concentrated using a 50 kDa Amicon tube at 4 °C, buffer exchanged into storage buffer (elution buffer plus 5% glycerol), and cryogenically frozen. For cellular applications, FMRP N-tat was diluted in PBS and bath applied to cells.

### Western blot

Western blots were performed using standard protocols. Briefly, cells were washed with ice-cold PBS and lysed in RIPA buffer supplemented with phosphatase and proteases inhibitors. Protein lysates were quantified by BCA protein assay, diluted in sample buffer (250 mM Tris-HCl pH 6.8, 8% SDS, 0.4% Bromophenol Blue, 40% glycerol, and 20% β-mercaptoethanol), resolved using 4 to 12% SDS-PAGE, and transferred to nitrocellulose membranes. Membranes were blocked in TBS-0.1% Tween with 5% skimmed milk for 1-h at RT, followed by overnight incubation with primary antibodies at 4 °C. Secondary antibodies were incubated for 1-h at RT. Primary and secondary antibodies are listed in Table S1. Protein detection was performed using Super Signal West (ThermoFisher). Immunoblots were imaged with a GE Amersham ImageQuant 800.

### RT-qPCR

Total mRNA was extracted from adherent cells using the RNeasy kit (Qiagen). Reverse transcription was performed using QuantiTect Reverse Transcription kit (Qiagen). Quantitative PCR was performed using Power Track SYBR Green Master mix (Applied Biosystems) following manufacturer instructions. Primer sequences are listed in Table S2.

### Immunofluorescence

For immunofluorescence (IF), cells were fixed with 4% PFA in PBS for 10 min at RT, followed by permeabilization with 0.2% Triton X-100 for 10 min at RT. Blocking was performed using 5% BSA in 0.05% Triton X-100 for 1-h at RT. HA primary antibodies were incubated overnight at 4 °C, and secondary antibodies were incubated for 1-h at RT. Following incubations, cells were stained with Hoechst (1:10,000) for 5 min at RT. Primary and secondary antibodies are listed in Table S1. Images were captured using an EVOS Cell imaging system.

### JC10 assay

Mitochondria membrane potential was measured with the JC10 assay kit (abcam) according to the manufacturer’s instructions. Briefly, tsA-201 and tsA-201 *FMR1*-KO cells were plated into 96-well plates at a density of approximately 5000 cells per well. Cells were transfected with 10, 50, or 100 nM FMRP N-tat (in PBS) added directly to the culture media and incubated for 18 h. To prepare the dye-loading solution, 50 μl of 100X JC-10 (Component A) was mixed with 5 ml of assay buffer A (Component B), and 50 μl of this solution was added to each well. The plate was incubated at 37 °C for 30 min, protected from light. After incubation, 50 μl per well of assay buffer B (Component C) was added to the plate prior to fluorescence measurement. Fluorescence intensities were recorded at excitation/emission wavelengths of 490/525 nm (cutoff at 515 nm) and 540/590 nm (cutoff at 570 nm) for ratio analysis.

### Immunoprecipitation

Cells were treated with FMRP N-tat (in PBS) or with PBS alone (negative control) for 6 h at 37 °C in complete media. Following treatment, both FMRP N-tat-treated and untreated cells were washed with PBS and crosslinked with DSP in PBS for 30 min at room temperature. Crosslinking was quenched by the addition of 20 mM Tris (pH 7.5) for 15 min at room temperature. Cells were lysed using ice-cold lysis buffer (25 mM Tris-HCl, pH 7.4; 150 mM NaCl; 1 mM EDTA; 1% NP-40; 5% glycerol) supplemented with protease inhibitors.

Lysates were pre-cleared by incubation with washed protein G magnetic beads (ThermoFisher) for 30 min at room temperature with gentle agitation. Cleared lysates were transferred to fresh tubes and incubated with HA antibody overnight at 4 °C with gentle agitation. The following day, protein G magnetic beads were added and incubated with the lysates for 2 h at 4 °C. Beads were then washed twice with lysis buffer and 10 times with PBS to remove residual detergent prior to submission for mass spectrometry analysis. All immunoprecipitation experiments were performed independently in triplicate, with each experiment including both FMRP N-tat-treated and untreated samples.

### LC-MS/MS

Samples were processed for MS at the Proteomics Core Facility at the Institute for Research in Immunology and Cancer (IRIC), Université de Montréal. Samples were reconstituted in 50 mM ammonium bicarbonate, 8M urea, vortexed and further diluted to 50 mM ammonium bicarbonate 1M urea with 10 mM TCEP [Tris(2-carboxyethyl) phosphine hydrochloride] (ThermoFisher), and vortexed for 1-h at 37 °C. Chloroacetamide (Sigma-Aldrich) to a final concentration of 55 mM, was added for alkylation. Samples were vortexed for 1 h at 37 °C. One microgram of trypsin was added, and digestion was performed for 8 h at 37 °C. Supernatants were dried down and solubilized in 5% ACN-4% formic acid. Samples were loaded on a 1.5 μl pre-column (Optimize Technologies). Peptides were separated on a home-made reversed-phase column (150-μm i.d. by 200 mm) with a 56-min gradient from 10 to 30% ACN-0.2% formic acid and a 600-nl/min flow rate on an Easy nLC-1200 connected to a Exploris 480 (ThermoFisher). Each full MS spectrum acquired at a resolution of 120,000 was followed by tandem-MS (MS-MS) spectra acquisition on the most abundant multiply charged precursor ions for 3 s. Tandem-MS experiments were performed using higher energy collision dissociation (HCD) at a collision energy of 34%. The data were processed using PEAKS X Pro (Bioinformatics Solutions) and a Human Uniprot database. Mass tolerances on precursor and fragment ions were 10 ppm and 0.01 Da, respectively. Fixed modification was carbamidomethyl (C). Variable selected posttranslational modifications were acetylation (N-ter), oxidation (M), deamidation (NQ), and phosphorylation (STY). The data were visualized with Scaffold 5.0 (protein threshold, 99%, with at least 2 peptides identified and a false-discovery rate [FDR] of 1% for peptides).

### Data analysis

Proteomics analyses were performed by the Center for Advanced Proteomics Analyses, a node of Canadian Genomic Innovation Network supported by Genome Canada. MS proteomics data were analyzed with STRING (string-db.org) and DAVID (28, 29). Western blots were quantified using Quantity One (BioRad). All data were analyzed using GraphPad Prism software. Where applicable, all data are represented by the mean (±sd) of independent experiments, and *p*-values were calculated using a one-tailed paired *t* test (as described in the figure legends) or two-tailed paired *t* test (as described in the figure legends). Microscopy images were prepared using Image J software. Display items were prepared using Adobe Illustrator and created with BioRender where indicated.

**Figure 2:**
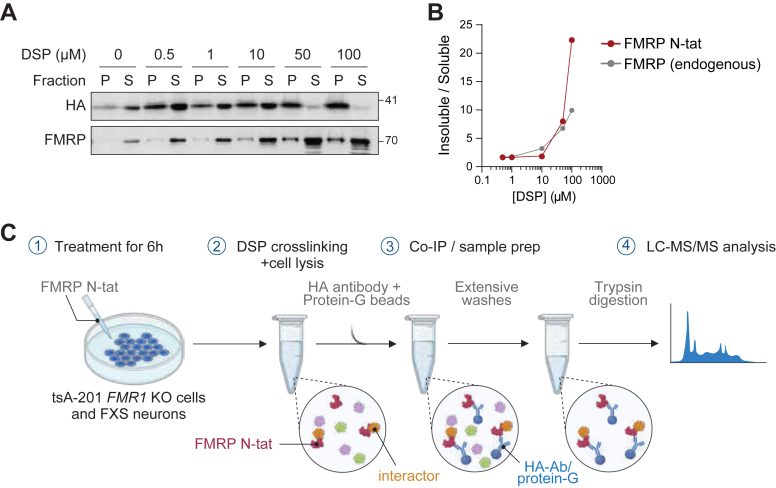
**Optimization of crosslinking and experimental approach for IP coupled to LC-MS/MS for FMRP N-tat**. *A*, DSP crosslinking was optimized in tsA-201 WT cells to take advantage of the interaction with endogenous FMRP. Immunoblot analysis of FMRP N-tat (detected by an HA-tag antibody) and endogenous FMRP (FMRP) in a soluble fraction (S) and insoluble pellet (P) isolated from cell lysates following DSP crosslinking. The DSP concentrations (μM) interrogated are indicated. *B*, quantification of the western blots (*panel A*) of FMRP N-tat (*red line*) and endogenous FMRP (*grey line*) represented as the ratio of protein found in the insoluble over soluble fractions. *C*, schematic of the experimental approach for AP-MS coupled to crosslinking for IP of FMRP N-tat in tsA-201 *FMR1* KO cells and FXS patient iPSC-derived neurons. Created with BioRender.

**Figure 3:**
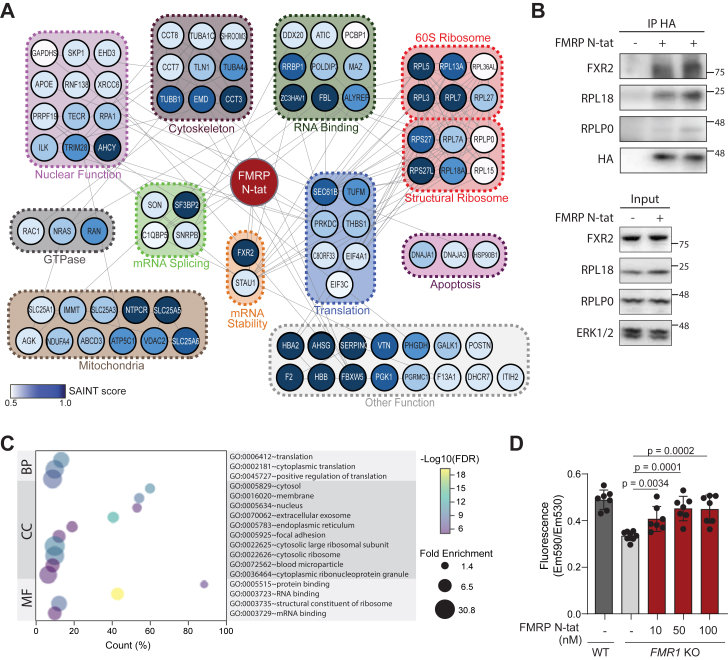
**Analysis of FMRP N-tat interacting proteins identified in tsA-201 *FMR1* KO cells**. *A*, FMRP N-tat associated proteins organized based on STRING analysis and functional annotations. *Circle color* intensity based on SAINT score. FMRP N-tat-associated proteins were functionally categorized based on GO molecular function terms and information compiled from NCBI Gene, GeneCards, and relevant literature sources. *B*, Co-immunoprecipitation and Western blot analysis of proteins associated with FMRP N-tat. Lysates from tsA-201 *FMR1*-KO cells treated with 100 nM FMRP N-tat were immunoprecipitated using anti-HA antibodies, followed by western blotting to detect the indicated associated proteins. Two biological replicates of the FMRP N-tat IP are shown. The negative control consisted of anti-HA IP from untreated cells (no peptide). Input lysates represent 10% of the total protein. Molecular weight markers are indicated. *C*, GO analysis of FMRP N-tat associated proteins identified. GO term biological process (BP), cellular compartment (CC), and molecular function (MF) are indicated. FDR was calculated from the −Log10 of the *p*-value. Fold enrichment indicated by the size of the circle. *D*, JC-10 analysis of mitochondrial membrane potential (MMP) in tsA-201 and tsA-201 *FMR1*-KO cells. *FMR1*-KO cells were treated with the indicated dose of FMRP N-tat for 18 h prior to analysis. MMP was assessed by calculating the fluorescence emission ratio (Em590/Em530). Each *dot* represents an independent biological replicate (n = 7). *p*-values were determined using a two-tailed student’s *t* test.

## Results

### Functional validation of a therapeutic FMRP protein

FMRP N-tat comprised the first 297 amino acids of human FMRP including the NDF (Agenet1/2), NLS, KH0/KH1 domains, and 17 amino acids of the KH2 domain, representing ∼50% of the full-length protein (Fig. 1*A*). His- and HA-tags at the N-terminus and a tat-tag at the C-terminus enabled purification, detection, and cell penetration, respectively. Following expression and purification of FMRP N-tat, examination of FPLC fractions by Coomassie gel and Western blot of the purified protein revealed a single protein product with minimal impurities (Figs. 1*B*, Fig. S1, *A*–*C*). The apparent molecular weight (MW) of 38,284 Da measured by QTOF-MS was consistent with the expected MW of the recombinant protein (Fig. S1*C*). The recombinant protein was stable, exhibiting minimal precipitation after freeze thaw and enabling concentrated samples (>1.0 mg/ml).

The recombinant protein was examined in cell-based assays for cellular uptake mediated *via* tat. Recombinant FMRP N-tat (50 nM) was applied directly in the growth media of HEK293 cells, and the cellular uptake analyzed by immunofluorescence (IF) 6-h post transfection with primary antibodies recognizing the HA-tag. The FMRP N-tat internalized protein was detected by faint but wide and diffuse cytoplasmic staining (Figs. 1*C*, S1*D*). In some cells, punctate nuclear localization was observed (Fig. 1*C*). Intracellular stability of FMRP N-tat was evaluated in HEK293 cells by adding it to the cellular media and measuring HA-tagged protein levels *via* Western blot over 48 h, normalized to vinculin. Based on the amount of FMRP N-tat at time zero (spike-in control) compared to the amount detected in the cell at the 2-h time points (Fig. S1*E*), approximately 75% of FMRP N-tat either failed to permeate the cell or had lost the HA-tag due to proteolytic cleavage. Quantification of FMRP N-tat following cellular uptake estimated an *in vitro* half-life of 9.1 h (Figs. 1*D*, S1*E*).

As a cell model for studying FXS, an isogenic tsA-201 cell line was generated using CRISPR to introduce a frameshift mutation in the *FMR1* locus, resulting in loss of FMRP expression (27). The tsA-201 cells are a derivative of HEK293 that stably express an SV40 temperature-sensitive (ts) T antigen. Interestingly, the HEK cell line is believed to originate from a neuronal lineage and shares more than 90% of its expressed genes with neuronal cells (30, 31, 32), making it a relevant model for studying FMRP N-tat interactions. Comparison of endogenous FMRP expression by Western blot in tsA-201 parental (WT) and tsA-201 *FMR1* knockout (*FMR1* KO) lines confirmed the knockout (Fig. 1*E*, lanes 1–4). Further, using an antibody that recognizes an N-terminal epitope of FMRP, the internalization of FMRP N-tat in tsA-201 *FMR1*-KO cells was found at levels higher than those of endogenous FMRP in tsA-201 WT cells (Fig. S1*F*).

FMRP modulates translation by regulating ribosome access to target mRNAs and affects mRNA stability, leading to changes in protein expression (15, 33), either upregulating or downregulating expression depending on the cell type (14, 34). Two previously reported FMRP regulated target mRNA transcripts, DLG4 (a.k.a. PSD95) and AKT (14, 15, 35), were interrogated for dysregulation at the protein level by Western blot in tsA-201 WT and tsA-201 *FMR1* KO cells. Both proteins exhibited an approximate 50% reduction in expression in the *FMR1* KO line compared to WT (Fig. 1*E*, lanes 1–4, Figs. 1*F*, S1*G*). Efficacy of FMRP N-tat to modify aberrant translation was demonstrated by applying FMRP N-tat (50 nM or 100 nM) on tsA-201 *FMR1* KO cells for 18 h followed by cell lysis and examination of changes in target protein expression. Addition of FMRP N-tat to cells caused increased expression of both DLG4 and AKT (Fig. 1, *E* and *F*), consistent with previous reports that endogenous FMRP can enhance the expression of proteins (14, 36). Western blot analysis further confirmed the cellular uptake of FMRP N-tat, as evidenced by HA detection in the treated *FMR1* KO cells (Fig. 1*E*, lanes 5, 6). These findings, consistent with previous reports (26, 27), demonstrated that FMRP N-tat can restore protein expression from two mis-regulated FMRP target mRNAs, suggesting rescue of lost function.

### Optimization and analysis of crosslinked AP coupled MS of FMRP N-tat

To understand the mechanism of action of FMRP N-tat in modulating lost FMRP function, affinity purification mass spectrometry (AP-MS) was performed using FMRP N-tat as the bait. It was hypothesized that FMRP N-tat should share some of the same molecular interactions in the interactome as endogenous FMRP (37, 38, 39, 40, 41). However, given that the FMRP N-tat lacked the C-terminus of the endogenous protein, it was unknown to what degree the FMRP N-tat protein would recapitulate a functional interactome.

It has been shown previously that the NDF of FMRP (amino acids 1–134) can direct dimerization with endogenous FMRP (20). Consistent with these observations, FMRP N-tat applied to the cellular media or overexpressed off a CMV-driven transgene in tsA-201 WT cells was able to IP endogenous FMRP (Fig. S2*A*). Therefore, to stabilize FMRP N-tat associated proteins, a crosslinking strategy for FMRP N-tat was developed using dithiobis-(succinimidyl propionate) (DSP) in tsA-201 WT cells, to take advantage of FMRP N-tat interaction with endogenous FMRP as a measure of IP effectiveness. DSP crosslinking was performed following a previously published protocol (42) and interrogated at a range of concentrations from 0.5 to 1.0 mM. Unfortunately, under these conditions, FMRP N-tat protein was primarily detected in an insoluble pellet, which as expected, contained endogenous FMRP (Fig. S2*B*, S2*C*). In the next experiment, the DSP concentration was lowered to 0.5 to 100 μM and again queried for FMRP N-tat in the soluble *versus* insoluble fractions (Fig. 2, *A* and *B*, Fig. S2*D*). At DSP concentrations between 1 and 10 μM, FMRP N-tat was found to be enriched in the soluble fraction, which was also enriched for endogenous FMRP (Fig. 2, *A* and *B*). Based on these results, 10 μM DSP was selected for crosslinking in subsequent experiments.

With the optimized DSP crosslinking established, tsA-201 *FMR1* KO cells were treated with FMRP N-tat, followed by DSP crosslinking, FMRP N-tat pull down using antibodies recognizing the HA-tag, and LC-MS (Fig. 2*C*). Given the *in vitro* half-life of FMRP N-tat (9.1 h; Fig. 1*D*), it was hypothesized that FMRP N-tat would rapidly engage cellular proteins upon entering the cytoplasm. Therefore, to identify interactions, DSP crosslinking was performed 6 h post-treatment, a time point when FMRP N-tat remained detectable in cells (Fig. S1*F*). IP experiments were performed from biological replicates (n = 3) of tsA-201 *FMR1* KO cells treated with 50 nM FMRP N-tat. The relative abundance of FMRP N-tat in the IP samples, compared to the negative control, was validated before conducting MS by western blotting of DSP-crosslinked samples following IP (Fig. S3*A*). The enrichment was further confirmed by increased total spectral counts measured by MS in treated cells compared to untreated controls (Fig. S3*B*).

Following LC-MS, a SAINT (Significance Analysis of INTeractome) score analysis was performed. SAINT estimates the likelihood of true interactions by comparing real and false interaction patterns and assigns confidence scores to the interaction data (43). A confidence score was applied using SAINT for each MS identified FMRP N-tat interactor (Table S3). A peptide untreated group was used as a negative control for optimal confidence in SAINT performance. Proteins with a SAINT score of 0.5 or higher were considered significant. From this analysis, 87 proteins were significantly enriched in tsA-201 *FMR1* KO cells treated with FMRP N-tat (Fig. 3*A*).

Assessed using a combination of STRING analyses and DAVID functional annotations, the FMRP N-tat interactome revealed enrichment in functional categories including RNA binding, RNA splicing and stability, translation, nuclear and mitochondria function, and structural constituents of the ribosome, which aligned with the known roles of FMRP (Fig. 3*A*). Several FMRP N-tat associated proteins, including FXR2, RPL18, and RPLP0, were validated by co-IP followed by Western blot analysis (Fig. 3*B*). Gene Ontology (GO) term analysis (Fig. 3*C*) revealed significant enrichment in functional categories of proteins involved in translation (*p* = 4.88E-08), cytosolic function (*p* = 1.98E-09), RNA binding (*p* = 5.56E-18), and constituents of the ribosome (*p* = 1.26E-07). These GO-captured terms suggested that FMRP N-tat was able to recapitulate many of the known roles of endogenous FMRP.

Based on the presence of mitochondrial-associated proteins in the FMRP N-tat interactome (Fig. 3*A*) and previous reports of mitochondrial membrane potential (MMP) defects in FXS cells (44, 45, 46, 47), it was hypothesized that FMRP N-tat might restore the impaired MMP phenotype. The MMP was assessed using the JC-10 assay, which detects a fluorescence shift from green to red as the dye accumulates in polarized mitochondria. Consistent with the literature, tsA-201 *FMR1*-KO cells had a defective MMP compared to WT cells (Fig. 3*D*). To evaluate rescue, tsA-201 *FMR1*-KO cells were treated with increasing concentrations of FMRP N-tat (10 nM, 50 nM, 100 nM), and MMP was measured. FMRP N-tat treatment resulted in a significant, dose-dependent restoration of MMP toward wild-type levels (Fig. 3*D*), supporting the functional relevance of mitochondrial proteins identified in the FMRP N-tat interactome. These findings suggested that FMRP N-tat can restore mitochondrial function lost in the absence of FMRP, thus validating predictions based on the IP-MS dataset.

### Identification of the FMRP N-tat interactome in FXS iPSC-derived neurons

**Figure 4:**
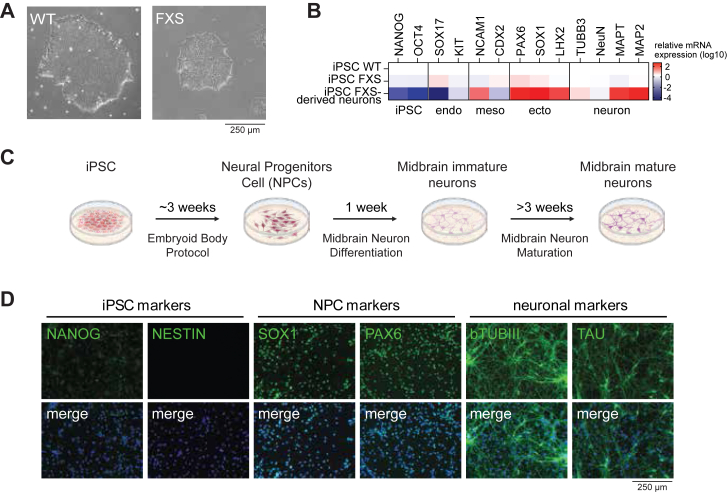
**Differentiation and characterization of FXS iPSCs and iPSC-derived midbrain neurons**. *A*, representative phase contrast images of WT and FXS iPSCs. *B*, gene expression analysis of the indicated markers measured by RT-qPCR in iPSC (WT and FXS) and FXS iPSC-derived neurons. Markers for iPSCs, endoderm (endo), mesoderm (meso) or ectoderm (ecto) lineages, and markers for mature neurons (neuron) are indicated. Values are log10 transformed and normalized to the iPSC WT cells. *C*, schematic representation of the protocol and timeline to differentiate iPSCs into midbrain mature neurons. Created with BioRender. *D*, IF analysis of the indicated lineage markers in FXS iPSC-derived neurons. Markers for iPSCs, neural progenitor cells (NPC) and neurons are indicated. Nuclear staining with DAPI.

To expand the limited understanding of functional FMRP-associated protein networks in human neurons, FMRP N-tat interactions were next examined in an FXS patient-derived induced pluripotent stem cell (iPSC) neuronal model. First, the FXS iPSCs were validated for *FMR1* expression, shown to display normal iPSC morphology and expressed the predicted iPSC markers (Figs. 4*A*, Fig. S4, *A* and *B*). FXS iPSCs were pluripotent as they effectively differentiated into the three germ layers, with the expected morphology and expression of the expected lineage markers, which were seemingly unaffected by the lack of *FMR1* expression (Figs. 4*B*, S4*C*).

FXS midbrain neurons were derived from FXS iPSCs using an embryoid body (EB) protocol, followed by midbrain neuronal differentiation and maturation (Fig. 4*C*). Neural induction involves patterning of iPSCs into the ectoderm lineage and differentiation into neuroectoderm giving rise to neural progenitor cells (NPCs). During the differentiation and maturation phase, additional patterning factors then drive the NPCs to midbrain neurons. Previously, defects in rosette formation during the EB protocol had been observed for FXS iPSCs (48). However, the FXS iPSCs used in the present study formed classic rosettes clear of any obvious defect (Fig. S4*D*). Following maturation of midbrain neurons, representative markers for the neuronal lineage were assessed by RT-qPCR and IF (Fig. 4, *B* and *D*). The FXS iPSC derived neurons had loss of iPSC markers (NANOG, NESTIN, and OCT4), concomitant with positive expression of ectoderm lineage markers (SOX1 and PAX6), and the expected neuronal specific markers (TUBB3, MAPT, and MAP2). Further, *FMR1* expression was not observed in FXS patient iPSC-derived NPCs and neurons compared to WT (Fig. S4*A*). These results validated the effective differentiation of FXS iPSCs into midbrain neurons enabling a FXS patient-derived model for identifying an FMRP N-tat interactome.

**Figure 5:**
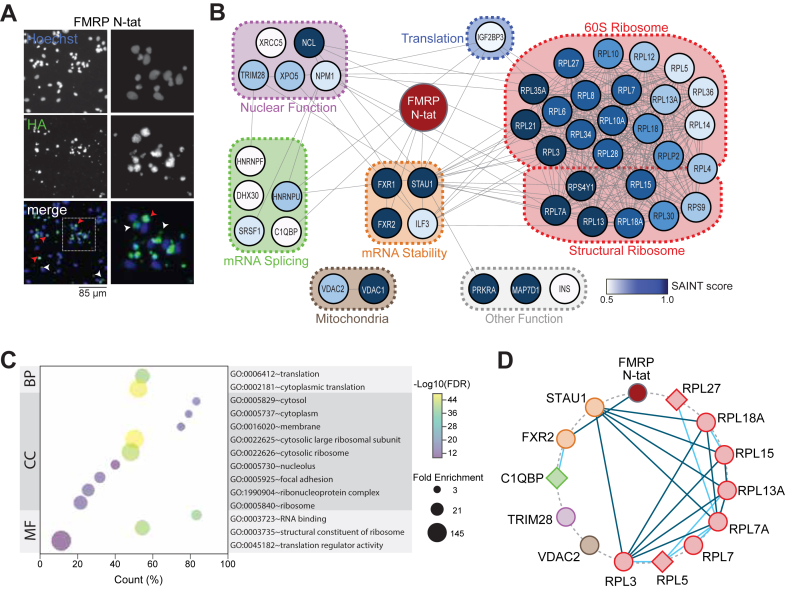
**Analysis of the FMRP N-tat interactome in FXS iPSC-derived midbrain neurons**. *A*, IF of FXS iPSC-derived neurons treated with FMRP N-tat (HA). FMRP N-tat was observed cytoplasmic (*white arrows*) and nuclear (*red arrows*). The dashed *white box* indicates the region shown in the zoomed-in panels on the right. Detection of FMRP N-tat by HA-antibody (*green*). Nucleus stained with Hoechst (*blue*). *B*, network of FMRP N-tat associated proteins in FXS iPSC-derived neurons based on STRING analysis and functional annotations. *Circle color* intensity is based on SAINT score. FMRP N-tat-associated proteins were functionally categorized based on GO molecular function terms and information compiled from NCBI Gene, GeneCards, and relevant literature sources. *C*, GO analysis of FMRP N-tat associated proteins in FXS iPSC-derived neurons. GO term biological process (BP), cellular compartment (CC), and molecular function (MF) are indicated. FDR was calculated from the -Log10 of the *p*-value. Fold enrichment indicated by the size of the circle. *D*, FMRP N-tat core proteins analyzed using BioPlex3.0. Bait proteins are represented by a *circle* and prey proteins with a *square*. Reciprocal interactions (*dark blue*) and one-way interactions (*light blue*) are represented by *colored lines*. Color of functional annotation as per *panel B*.

FMRP N-tat internalization *via* tat was confirmed in neurons using IF of treated *versus* non-treated cells (Figs. 5*A*, Fig. S5*A*). FMRP N-tat, as evidenced by HA-tag immune staining, was effectively taken up by neurons, localized primarily to the cytoplasm with some nuclear distribution (Fig. 5*A*, compare white and red arrows). AP-MS of FMRP N-tat treated FXS neurons was conducted as described previously for tsA-201 *FMR1* KO cells. Briefly, FMRP N-tat (50 nM) was applied in the neuronal growth media for 6 h, followed by DSP crosslinking, IP of FMRP N-tat complexes, and LC-MS. IP experiments were performed from biological replicates (n = 4) of FMRP N-tat treated FXS neurons. FMRP N-tat protein abundance was verified in FXS neuronal lysates, found enriched in DSP crosslinked lysates following IP as confirmed by Western blot and in enriched in the total spectral counts by MS (Fig. S5*B*, S5*C*). A SAINT score cutoff of 0.5 or higher was again considered significant. Overall, 48 high confidence FMRP N-tat associations were identified (Fig. 5*B*, Table S4).

Using a combination of STRING analysis and DAVID functional classification, the FMRP N-tat associations in FXS neurons were generally like those observed in tsA-201 *FMR1* KO cells, found to be associated with RNA stability, mRNA splicing, mitochondrial function, translation, and structural constituents of the ribosome (Fig. 5, *B* and *C*). Interestingly, of the 48 proteins identified, 44 could be assigned to RNA binding functions and included ∼30% (25 of 78) of the known 40S/60S ribosomal proteins. GO term analysis (Fig. 5*C*) revealed significant enrichment of processes involved in translation (*p* = 5.95E-38), cytosolic ribosome localization (*p* = 4.44E-41), RNA binding (*p* = 2.83E-37), and structural constituents of the ribosome (*p* = 6.86E-39). In total, 13 proteins identified in FXS neurons were also identified in tsA-201 *FMR1* KO cells (Fig. S5*C*). Using BioPlex (biophysical interactions of ORFeome-based complexes) Network 3.0 (49, 50), the 13 common factors were analyzed against previously validated protein–protein interactions, reinforcing the involvement of key proteins in essential FMRP functions (Figs. 5*D*, S3*C*). These proteins included previously identified *bona fide* FMRP interactors FXR2, STAU1, TRIM28, VDAC2, and C1QBP known to regulate mRNA stability, stress responses, and mitochondrial function (Fig. 5*D*), and ribosomal proteins, several of which have been previously identified to interact with the N-terminus of endogenous FMRP (37).

**Figure 6:**
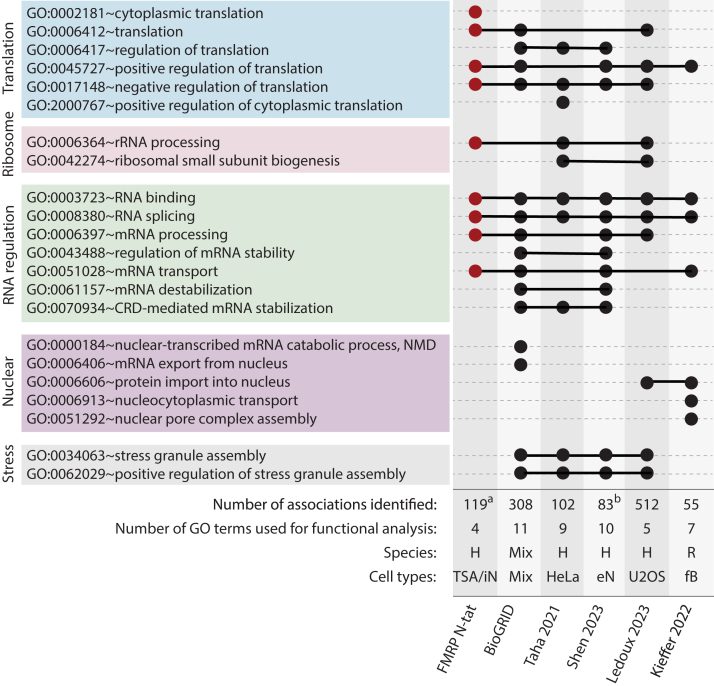
**Analysis of GO term functional associations related to endogenous FMRP**. Dumbbell plot analysis of GO biological processes associated with proteins interacting with FMRP N-tat compared to those interacting with endogenous FMRP or fragments of FMRP as identified from the BioGRID database or reported in the indicated publications. *Colored boxes* provide a high-level categorization of the various molecular functions of FMRP derived from the indicated GO terms. The BioGRID data are compiled from 150 published studies. Taha 2021 (37) analyzed three fragments: N-terminal (1–218), middle (212–425), and C-terminal (444–632); Shen 2023 (52) examined full-length FMRP from human neurons; Ledoux 2023 (51) investigated nuclear isoform 6, which excludes exon 12 located in the C-terminal region of full-length FMRP; Kieffer 2022 (38) examined an N-terminal fragment (residues 1–213) in nuclear preparations. The number of associations refers to the FMRP protein–protein interactions identified in each study. The number of GO terms used for functional analysis was determined by selecting the top significant terms (*p* < 10E-08) from each study. (a) Proteins associated with FMRP N-tat identified in AP-MS experiments using both tsA-201 cells and iPSC-derived neurons, (b) Includes only FMRP associated proteins showing >2-fold enrichment. eN, embryonic neurons; fB, forebrain; H, human; iN, iPSC derived neurons; Mix, mixed; R, rat; TSA, tsA-201.

To assess the fidelity of molecular function rescue by FMRP N-tat in iPSC neurons and tsA-201 *FMR1* KO cells, GO term analysis was used to compare functional processes regulated by FMRP N-tat to those associated with endogenous FMRP and other N-terminal fragments, as reported in previous studies (37, 38, 51, 52). GO terms related to biological processes and showing high statistical significance (*p* < 10E-08), were extracted from each study to identify terms commonly associated with N-tat across individual studies and the BioGRID dataset. For positive inclusion, GO terms were considered commonly associated only if they were significantly enriched (*p* < 0.01) in compared datasets. The dumbbell plot analysis (Fig. 6) summarizes the results and demonstrated that FMRP N-tat can recapitulate key processes related to translation, ribosomal processing, and RNA regulation. Although nuclear molecular functions were observed for endogenous FMRP, GO analysis did not identify these processes as significantly enriched for FMRP N-tat, despite the presence of several nuclear proteins in the IP-MS dataset (Figs. 6, Fig. 3*A*, 5*B*, Table S3). Collectively, FMRP N-tat may restore lost functional activity in FXS cell models by engaging relevant protein factors.

## Discussion

In the present study, tsA-201 *FMR1* KO cells and iPSC-derived neurons of patients with FXS revealed the potential of an FMRP N-tat therapeutic for protein replacement in FXS. The results show that the N-terminal fragment of FMRP, encompassing residues 1 to 297 of the endogenous protein, was efficacious to recapitulate protein interactions regulating mRNA splicing, RNA stability, mitochondrial function, translation, and constituents of the ribosome to restore lost functions observed in FXS.

The ability to deliver protein payloads intracellularly to tissues or organs is highly desirable and achievable with cell-penetrating peptides such as tat (2, 53, 54). Initially, longer tat-tag sequences were shown to impart some element of toxicity (53, 54), however, the tat-tag sequence used with FMRP N-tat constituted residues 48 to 57, which permitted efficient translocation through the plasma membrane, strong retention once internalized, and low toxicity (24). Indeed, FMRP N-tat displayed no apparent toxicity *in vitro* or *in vivo* and was shown to effectively cross the BBB and cross cellular membranes into neurons in the cerebellum and hippocampus (26).

The misregulation of FMRP target mRNAs has been shown to involve the translation machinery, predominantly the 60S large ribosomal subunit (55), which correlated with data presented here for FMRP N-tat. Specifically, loss of FMRP in FXS disrupts FMRP directed ribosome recruitment to FMRP regulated transcripts (21, 55, 56, 57). Interestingly, the FMRP N-tat fragment containing only KH0 and KH1 RNA recognition motifs and short piece of KH2, can restore many aspects of full-length FMRP function, including regulation of translation, ribosomal processing, and RNA regulation. Both the KH0 and KH1 domains have been shown to contain essential mRNA binding activity (17, 58). Previously, the two RNA-binding domains, KH1 and KH2 of FMRP have been shown to recognize distinct and independent RNA motifs (35). Further, specific binding sites within mRNA targets for both WT and the I304N mutant FMRP isoforms have been identified, indicating that transcript recognition can occur in a selective and domain-specific manner. Together, these findings support the idea that protein replacement of FMRP may not require the presence of all three RNA-binding domains (KH0, KH1, and KH2) but that individual domains or fragments may retain meaningful biological activity.

One of the few studies examining FMRP interactions specifically in human neurons used multi-omics analyses to identify FMRP-interacting proteins in *postmortem* mid-fetal human cortical tissue (52). The study demonstrated that FMRP interacts with CNOT1 (CCR4-NOT Transcription Complex Subunit 1) involved in mRNA turnover and translation control, to maintain levels of receptor for activated C kinase 1 (RACK1), a key regulator of mitochondrial function. Reduction in RACK1 expression led to both mitochondrial dysfunction and neuronal hyperexcitability, phenotypes reminiscent of those observed in FXS neurons. Several independent studies have also reported mitochondrial phenotypes in FXS cells (44, 45, 46, 47), including defects in MMP, consistent with findings that FMRP N-tat can rescue MMP abnormalities. In addition, human neurons infected with lentiviral-sh*FMR1* exhibited increased mitochondrial fragmentation and elevated oxidative stress, further supporting the role of FMRP in maintaining mitochondrial integrity (52). Collectively, these findings highlight mitochondrial dysfunction as a key feature of FXS and suggest it may be a promising therapeutic target.

A previous study has performed AP-MS analyses with endogenously expressed fragments of N-terminal, central, and C-terminal FMRP in HeLa cells (37). The study identified FMRP binding proteins that corroborated FMRP function in RNA metabolism, translation, ribosome biogenesis, mitochondrial organization, and other diverse biological processes, aligning closely with the molecular functions of the FMRP N-tat associated proteins reported in the present study (Figure 6). Given that FMRP N-tat contained only the N-terminus of the endogenous protein, likely some protein interactions would not be possible using a truncated protein. Although the exact function of the NDF *in vivo* is still unclear, it has been reported as the site directing the majority of FMRP interactions (59).

Of the 180 factors observed in HeLa interactome, 28 interacted specifically with the N-terminus (amino acids 1–281) and 48 interacted with both the N- and C-terminus (37). The HeLa FMRP interactome confirmed known FMRP-binding proteins, including ribosomal proteins and found novel FMRP interacting proteins such as TRIM28, to implicate FMRP in multiple cellular processes including normal and cell stress conditions in neuronal as well as non-neuronal cell types (37).

Recently, another study performed AP-MS to identify nuclear proteins associated with an N-terminal fragment of FMRP (amino acids 1–213) in rat forebrain lysates (38). The study identified 55 protein candidates interacting with the NDF, including factors involved in chromatin remodeling, transcriptional regulation, and mRNA splicing. In the present study, several nuclear factors were identified (Figs. 3*A* and 5*B*), but only FXR1 was common to the nuclear proteins identified in the rat forebrain lysates. Since FMRP is mainly cytoplasmic at steady state with an estimated 2 to 4% endogenous FMRP present in the nucleus (60), may explain the discrepancy between datasets.

Endogenous FMRP has been reported to interact directly with its two close paralogues FXR1 and FXR2, and interactions with NUFIP1, CYFIP1, and CYFIP2 have also been demonstrated (40, 41). The interaction of FMRP N-tat with both FXR1 and FXR2 was observed in FXS iPSC-derived neurons in the present study; however, although FXR1 was found in tsA-201 *FMR1* KO cells treated with FMRP N-tat it did not reach the SAINT score cutoff. CYFIP1 or CYFIP2 were not identified in the present study; however, a previously described CYFIP1 interactor called Rac1 (61), a small GTPase, was identified to interact with FMRP N-tat in tsA-201 *FMR1* KO cells. The Rac1 pathway has been shown to be important in neuronal maturation and implicated in development and maintenance of neuronal structures (40, 62).

In addition to factors described earlier, the overlap of FMRP N-tat associated proteins observed in tsA-201 *FMR1* KO cells and FXS patient-derived neurons, pointed to C1QBP, TRIM28, STAU1, and VDAC2 as factors supporting functional rescue, all of which have previously been identified to interact with FMRP or have a role in FXS (37, 63). C1QBP (complement C1q binding protein) is a protein that is involved in many biological processes, including inflammation, infection, and mitochondrial function and known to interact with FMRP (37). TRIM28 (tripartite motif-containing 28) is a nuclear protein that is involved in cell growth, differentiation, and DNA repair, identified to interact with FMRP perhaps in regulation of stress responses, although its role in FXS is unknown (37).

STAU1 (Staufen double stranded RNA binding protein 1) is a known regulator of translation and mRNA decay important for neuronal differentiation (64). STAU1 regulates a mRNA decay pathway called Staufen-mediated mRNA decay (SMD) to degrade mRNAs that contain a translation termination codon upstream of a splicing-generated exon junction complex, thus protecting cells from proteins arising from abnormal mRNAs (65). SMD, and the related decay pathway nonsense mediated decay (NMD), both rely on an RNA helicase called UPF1 for RNA decay (66, 67). STAU1 binds to UPF1 to initiate SMD, and recently it was observed that FMRP interacts with UPF1 to mediate NMD (66). Loss of FMRP as observed in FXS, leads to hyperactivated NMD and inhibition of neuronal differentiation. The identification of STAU1 as an FMRP N-tat associated protein suggests mechanistic potential in an unexplored area of FXS research.

VDAC2 (voltage-dependent anion channel 2) protein plays a role in regulating cell metabolism and mitochondrial apoptosis. In FXS, deregulation of ER-mitochondria contact formation and mitochondrial calcium homeostasis is mediated by VDAC proteins (63). The C-terminus of FMRP was shown to facilitate the interaction, and expression of the C-terminus of FMRP (amino acids 423–632) was able to restore ER-mitochondria contact site formation and mitochondrial calcium homeostasis in FXS patient iPSC-derived neurons. Additionally, the C-terminal fragment alleviated locomotion and cognitive deficits in *Fmr1* KO mice. In the present study, it was demonstrated that FMRP N-tat can rescue the leaky MMP observed in FXS cells. The findings highlight key mitochondrial function possibly mediated by a VDAC2-FMRP interaction in FXS that can be restored by FMRP N-tat.

Past work has shown co-IP between FMRP N-tat with specific ion channel proteins that include Cav3.1 and Kv4.3 (26, 27), and FMRP interaction with slack potassium channels (68). FMRP N-tat IP did not recover Cav3.1 or Kv4.1 from either treated tsA-201 *FMR1* KO cells or FXS iPSC-derived midbrain neurons. The result is not unexpected, as neither cell type is known to endogenously express these ion channels. HEK-derived tsA-201 cells lack expression of these genes entirely, and studies with Cav3.1 and Kv4.1 are routinely performed using overexpression systems (27). Also, co-IP between FMRP and Kv1.2 is dependent on Kv1.2 phosphorylation, complicating detection (69). Further, these channels are typically restricted to specific neuronal subtypes such as nociceptive neurons, terminal ganglion cells, and interneurons (70). In contrast, the iPSC-derived midbrain neurons are not expected to express Cav3.1 or Kv4.1. Thus, the absence of these proteins in the IP-MS dataset likely reflects their limited or absent expression in the experimental models used, rather than a failure of the FMRP N-tat construct to interact with them.

Since the majority of FMRP interactions have been mapped to the NDF, an N-terminal fragment of FMRP may be sufficient to restore mis-regulation of target mRNAs observed in FXS. In addition, previous research has established the longest recombinant fragment of FMRP resistant to degradation and precipitation included amino acids 1 to 280, to the end of the KH1 domain, suggesting a formulation optimal therapeutic could be developed (71). However, a thorough investigation into additional fragments of FMRP, either arising from truncating the N-terminus or extending the C-terminus into the KH2 domain has yet to be performed.

Currently, no disease-modifying treatments for FXS exist to correct imbalances in protein synthesis. In the present study, it was observed that FMRP N-tat interacts with proteins that direct RNA stability and translational regulation. Further, FMRP N-tat can restore protein levels in the cerebellum of murine models of FXS and the interactome analysis suggests FMRP N-tat interacts with cellular machinery directing lost function in FXS patient iPSC-derived neurons. These results highlight the potential of tat-conjugated FMRP therapeutic protein as a tractable strategy for treatment of FXS.

## Data availability

All data are contained within the manuscript.

## Supporting information

This article contains supporting information.

## Conflict of interest

The authors declare the following financial interests/personal relationships which may be considered as potential competing interests: Dr R. W. Turner is inventor of a patent application on FMRP N-tat and its use as a therapeutic filed by UTI Limited Partnership (PCT/CA2019/000,001, 1/10/2019). The other authors declare no conflict of interest.
