# Supplementary material for: Interactome of FMRP-N-tat therapeutic unveils key interactions for cellular function in Fragile X neurons
Source: J Biol Chem. 2025 Jun 4;301(7):110341. doi: 10.1016/j.jbc.2025.110341 (PMC12246597; doi:10.1016/j.jbc.2025.110341)
Supplement: Supplemental Figures [file mmc1.pdf]

Figure S1

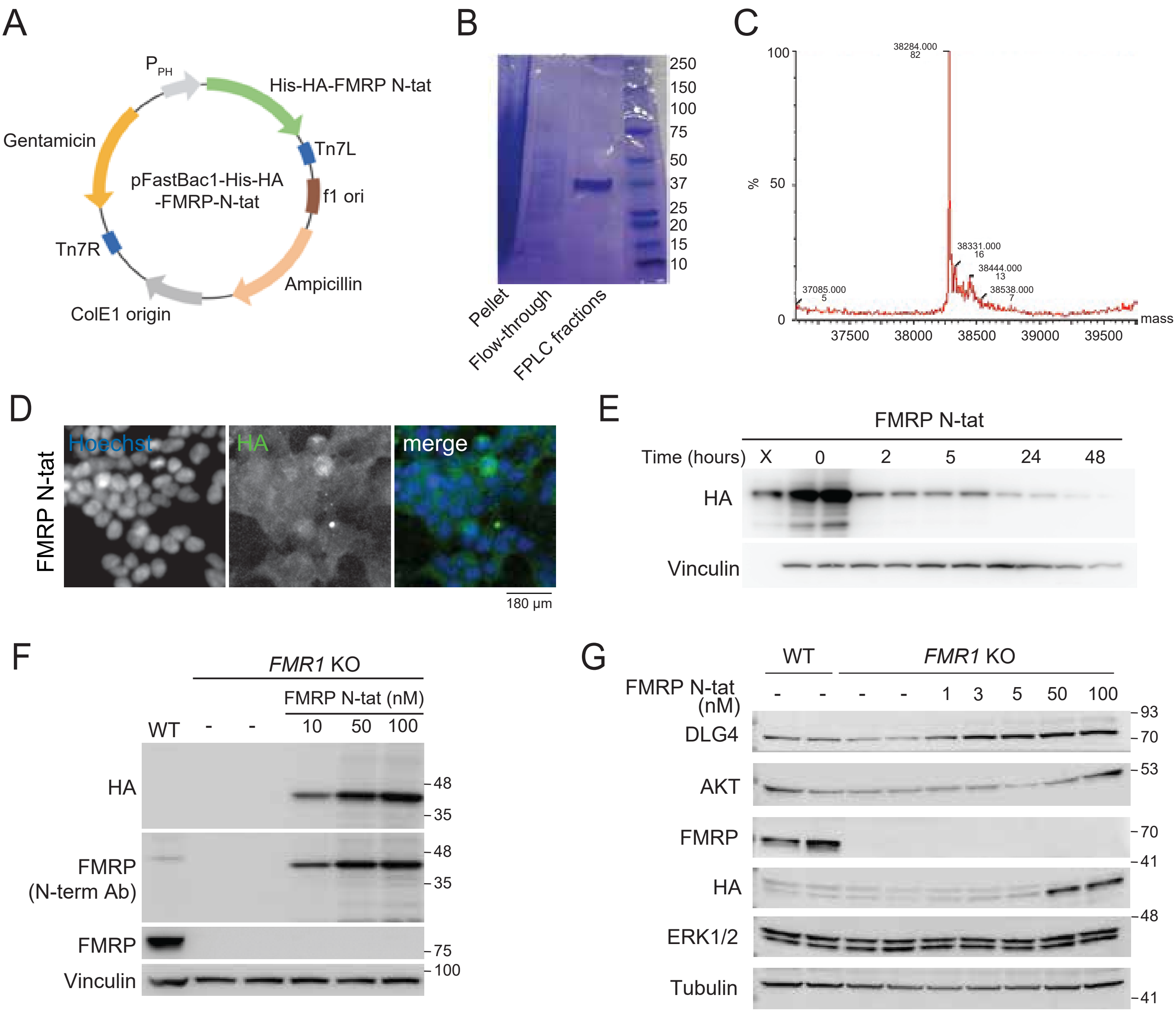

(**A**) Schematic representation of pFastBac-His-HA-FMRP-N-tat plasmid used for FMRP N-tat expression in insect cells.
(**B**) Analysis of immobilized metal affinity chromatography purified FMRP N-tat by SDS-PAGE (FPLC fractions). Pellet fraction and flow-through are included as controls. Gel stained by Coomassie blue.
(**C**) MW examination of purified FMRP N-tat by QTOF Mass Spectrometry.
(**D**) Higher magnification of immunofluorescence of HEK293 cells treated with FMRP N-tat presented in Figure 1C. FMRP-N-tat was added to the culture medium at 50 nM and its uptake confirmed using an anti-HA antibody (green) 18 hours post transfection. The blue signal corresponds to a nuclear stain (Hoesche).
(**E**) Evaluation of FMRP N-tat stability by western blot after addition of 50 nM FMRP N-tat to HEK293 cell culture media. Cells were maintained in culture for the indicated times. Each pair of lanes is an independent experiment. The time “X” represents an aliquot of FMRP N-tat from the stock solution. For time 0, FMRP N-tat was spiked into the cellular lysate. Westerns were probed with anti-HA antibody. Vinculin was used as a loading control to quantify FMRP N-tat expression to calculate the half-life of FMRP N-tat shown in Figure 1D.
(**F**) Western blot analysis of FMRP N-tat expression in FMR1-KO tsA-201 cells. Expression of endogenous FMRP in wild-type (WT) tsA-201 cells is shown. FMRP N-terminal (N-term) antibody (Ab) from Millipore MAB2160 and FMRP C-terminal antibody (FMRP) from CST#4317.
(**G**) Dose response of FMRP N-tat treatment (nM) in tsA-201 WT or tsA-201 FMR1-KO cells. Images are the uncropped membranes shown in Figure 1E.

Figure S2

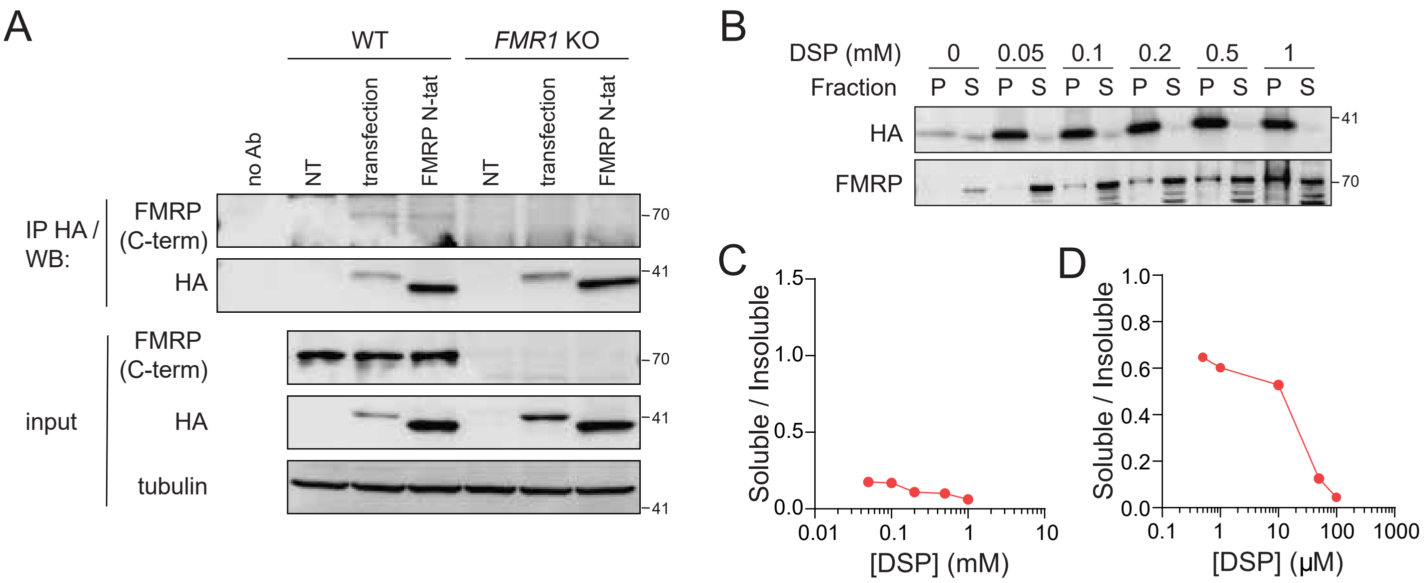

(**A**) Immunoprecipitation of purified bath applied FMRP N-tat (FMRP N-tat) or overexpressed off a CMV driven transgene (transfection) in tsA-201 WT or FMR1 KO cells. Non-treated (NT) cells were used as a control. Endogenous FMRP detected by a C-terminal specific antibody was co-immunoprecipitated in tsA-201 WT cells. FMRP N-tat is detected by antibody recognizing the HA-tag (HA). Tubulin was a loading control.
(**B**) Immunoblot analysis of FMRP N-tat and endogenous FMRP in soluble (S) and insoluble (pellet, P) fractions isolated following DSP crosslinking. DSP concentrations interrogated for crosslinking are indicated. FMRP marks the endogenous full-length protein and FMRP N-tat is identified by an antibody to the HA tag.
(**C**) Quantification of FMRP N-tat inclusion calculated from the ratio of soluble over insoluble fractions identified in the immunoblot shown in panel (B).
(**D**) Quantification of FMRP N-tat inclusion calculated from the ratio of soluble over insoluble fractions from the immunoblot shown in the main Figure 2A.

Figure S3

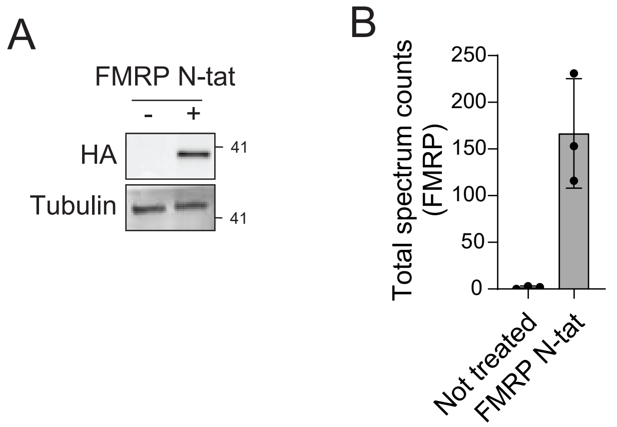

(**A**) Immunoblot of FMRP N-tat from lysates of control (-) or treated (+) tsA-201 FMR1 KO cells. Tubulin was used as a loading control.

(**B**) Verfication of FMRP N-tat protein abundance in DSP-crosslinked IP samples subjected to MS. Total spectrum counts for FMRP detected by MS in control cells (not treated) or cell treated with FMRP N-tat. Each dot represents an independent experiment (n=3).

Figure S4

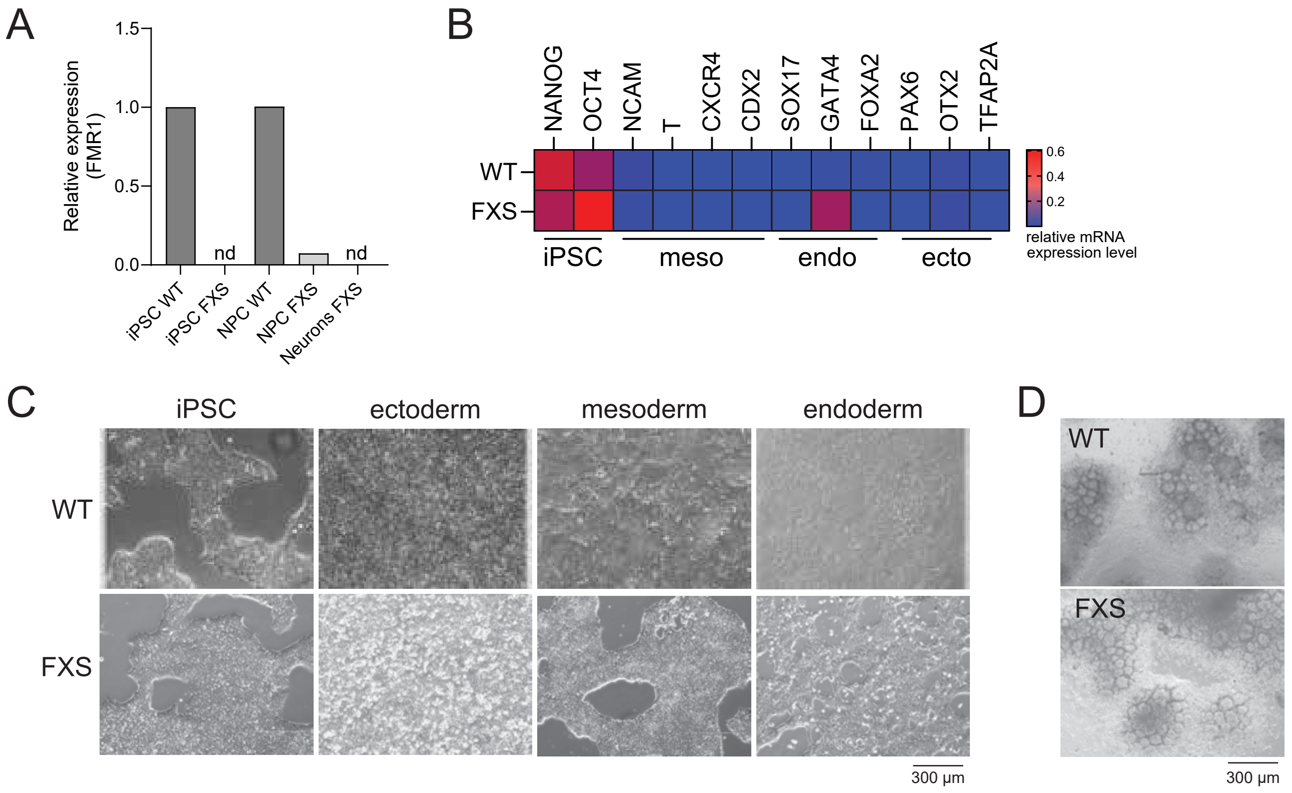

(**A**) FMR1 mRNA expression levels measured using RT-qPCR in indicated cell types. Expression was normalized to iPSC WT.
(**B**) Expression analysis of the indicated mRNA markers measured in WT iPSC and FXS patient iPSC by RT-QPCR. Markers for iPSCs, endoderm (endo), mesoderm (meso) or ectoderm (ecto) lineages are indicated. Expression levels are color coded (red high, blue low) as indicated.
(**C**) Representative phase contract images of WT- iPSC and FXS patient iPSC following differentiation via a trilineage assay. Shown are the resulting phenotypic changes for differentiation into each of the three germ layers: ectoderm, mesoderm and endoderm, relative to the starting iPSC morphology.
(**D**) Representative phase contrast images of WT- iPSC and FXS patient iPSC derived NPCs taken during rosette formation in the EB protocol for neuronal differentiation.

Figure S5

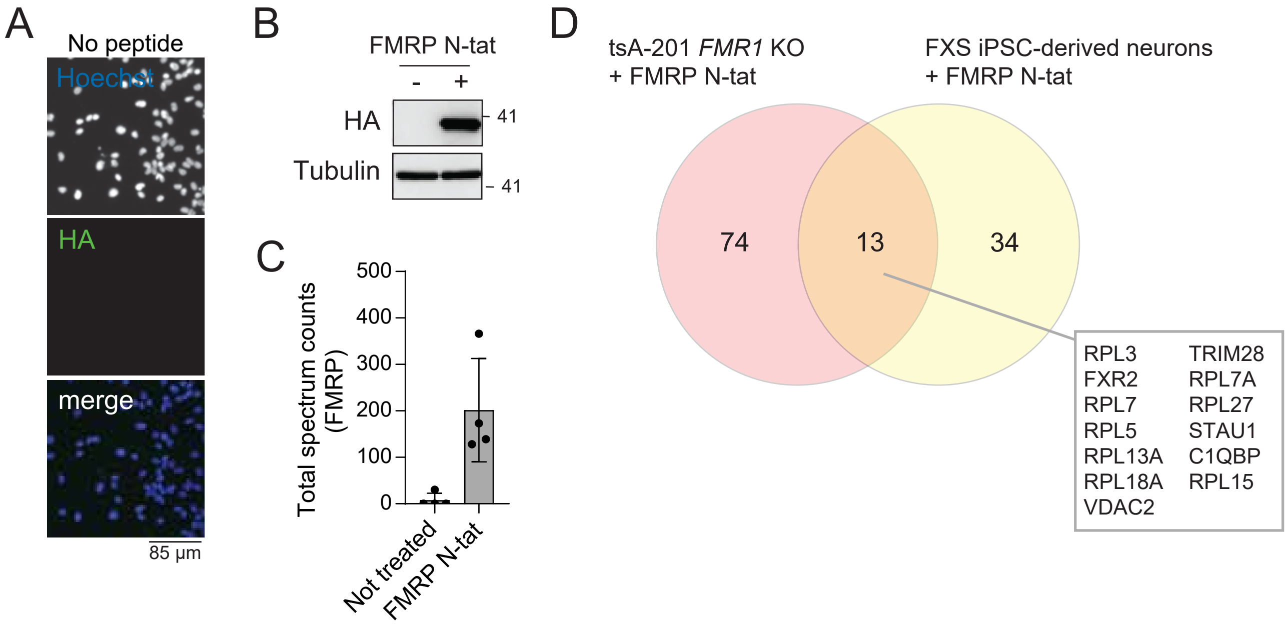

(**A**) IF of FXS iPSC-derived neurons treated with peptide control for the FMRP N-tat internalization experiment shown in Figure 5A. Detection of FMRP N-tat by HA-antibody (green). Nucleus stained with Hoechst (blue).

(**B**) Immunoblot of FMRP N-tat from lysates of control (-) or treated (+) FXS iPSC-derived neurons. Tubulin was used as a loading control.

(**C**) Verfication of FMRP N-tat protein abundance in DSP-crosslinked IP samples subjected to MS. Total spectrum counts for FMRP detected by MS in control cells (not treated) or cell treated with FMRP N-tat. Each dot represents an independent experiment (n=4).

(**D**) Venn diagram representing the overlap of FMRP N-tat interactors between tsA-201 *FMR1* KO and FXS iPSC-derived neurons.
