# Supplementary material for: Interactome of FMRP-N-tat therapeutic unveils key interactions for cellular function in Fragile X neurons
Source: J Biol Chem. 2025 Jun 4;301(7):110341. doi: 10.1016/j.jbc.2025.110341 (PMC12246597; doi:10.1016/j.jbc.2025.110341)
Supplement: Supplemental Tables [file mmc2.pdf]

Supplementary Table 1
Antibodies used for western and IF

Target	MW	Species	Cat.#	Vendor
AKT	62	Rabbit	#9272	Cell Signaling
β-tubulin	50-55	Mouse	sc-69969	SantaCruz
DLG4	95	Rabbit	#3450	Cell Signaling
ERK1/2	42/44	Rabbit	#9102	Cell Signaling
FMRP	78	Rabbit	#4317	Cell Signaling
FMRP (N-term)	78	Mouse	MAB2160	Millipore
FXR2	72-80	Rabbit	7098	Cell Signaling
HA	n/a	Rabbit	#3724	Cell Signaling
Nanog	35-40	Rabbit	PA1-097	Invitrogen/ThermoFisher
Nestin	200	Mouse	14-5843-82	Invitrogen/ThermoFisher
Pax6	46-48	Rabbit	60433S	Cell Signaling
RPL18	23	Rabbit	A10720	Abclonal
RPLP0	36	Rabbit	A13633	Abclonal
Sox1	39-42	Goat	AF3369	R&D systems
Tau	45-65	Mouse	5A6	Developmental Studies Hybridoma Bank
Vinculin	117	Rabbit	#13901	Cell Signaling
α-Mouse IgG HRP	n/a	Mouse	31430	Fisher Scientific
α-Rabbit IgG HRP	n/a	Rabbit	31460	Fisher Scientific
α-Mouse Alexa Fluor 594	n/a	Donkey	A-21203	ThermoFisher
α-Rabbit Alexa Fluor 488	n/a	Donkey	A-21206	ThermoFisher
α-Goat Alexa Fluor 800	n/a	Donkey	A32930	ThermoFisher

n/a = not applicable

Supplementary Table 2

Primers used to measure mRNA expression levels by RT-qPCR

Target	Forward primer	Reverse primer
FMR1	TCAAGGAATGGGTCGAGGTA	GCAGGAAGCTCTCCCTCTCT
NANOG	CAATGGTGTGACGCAGGGAT	GGACTGGATGTTCTGGGTCTG
OCT4	GGTGGAGAGCAACTCCGAT	TGCAGAGCTTTGATGTCCTG
SOX17	TGGACCGCACGGAATTTGAA	GCTGTCGGGGAGATTCACAC
KIT	TGGGAAGATTATCCCAAGTCTG	TGTGTAAGTGCCTCCTTCGG
NCAM1	AGAACGACGAGGCTGAGTACA	TTCCATGGCAGTCTGGTTCT
CDX2	CGGCAGCCAAGTGAAAACC	CTCCGGATGGTGATGTAGCG
PAX6	GGTTGGTATCCGGGGACTT	TCCGTTGGAACTGATGGAGT
SOX1	AAATACTGGAGACGAACGCCG	AACCCAAGTCTGGTGTCAGC
LHX2	CAAGGACTTGAAGCAGCTCG	TAAGAGGTTGCGCCTGAACT
TUBB3	TATCCCGACCGCATCATGAA	GGTCTCATCCGTGTTCTCCA
NeuN	CTCCGACCCTACAGAGAAGC	CGAATTGCCCGAACATTTGC
MAPT	GACCTCCAAGTGTGGCTCAT	GGACGTGGGTGATATTGTCC
MAP2	AGGGCTGGTAGGTTGGATCT	TGTGTCTCTGCCTTTGCATC

Supplementary Table 3

Significant interactors (SAINT > 0.5) in TsA201 *FMR1* KO cells treated with FMRP N-tat

Genes	Counts (1-297)	Counts (Not treated)	AvgP	MaxP	SAINT Score	Fold Change
RPS27L	7|11|3	0|0|0	1	1	1	70
AHSG	16|7|9	0|0|1	1	1	1	32
RPL3	19|29|21	1|4|5	1	1	1	6.9
ZC3HAV1	3|13|8	0|0|0	1	1	1	80
HBA2;HBA1	16|37|16	2|0|0	1	1	1	34.5
SLC25A5	29|30|26	8|7|9	1	1	1	3.54
SERPINC1	4|3|7	0|0|0	1	1	1	46.67
HBB	5|3|5	0|0|0	1	1	1	43.33
F2	8|7|11	0|0|0	1	1	1	86.67
FXR2	55|59|53	20|21|11	0.99	1	0.99	3.21
FBL	3|8|3	0|0|0	0.99	1	0.99	46.67
NTPCR	4|4|2	0|0|0	0.98	1	0.98	33.33
CCT3	5|3|2	0|0|0	0.98	1	0.98	33.33
SF3B2	2|3|3	0|0|0	0.98	0.99	0.98	26.67
AHCY	9|5|2	0|0|0	0.98	1	0.98	53.33
FBXW5	2|2|4	0|0|0	0.97	1	0.97	26.67
RPL7	21|16|15	7|5|2	0.96	1	0.96	3.71
RPL5	10|8|15	0|0|3	0.96	0.99	0.96	11
EMD	8|8|10	2|1|3	0.95	0.99	0.95	4.33
SLC25A6	18|17|19	0|0|7	0.94	0.95	0.94	7.71
TUBB1	17|7|15	0|0|4	0.92	0.99	0.92	9.75
RPS27	9|23|8	1|3|3	0.92	1	0.92	5.71
RPL13A	5|18|5	2|1|0	0.91	1	0.91	9.33
VTN	2|5|6	0|0|1	0.9	1	0.9	13
RRBP1	2|7|7	0|1|0	0.9	1	0.9	16
SEC61B	3|6|2	0|1|0	0.88	1	0.88	11
PGK1	5|5|4	0|2|0	0.85	0.88	0.85	7
RPL18A	7|10|10	2|2|3	0.84	0.98	0.84	3.86
VDAC2	7|3|6	0|0|2	0.84	0.97	0.84	8
PHGDH	9|8|4	0|0|3	0.83	0.95	0.83	7
TUFM	10|5|9	0|4|0	0.81	0.92	0.81	6
TRIM28	6|6|7	2|2|1	0.81	0.91	0.81	3.8
ATP5C1	6|2|2	0|0|1	0.8	1	0.8	10
TUBA4A	30|21|31	0|0|18	0.79	0.9	0.79	4.56
ALYREF	3|4|7	0|2|0	0.79	0.97	0.79	7
RAN	5|3|4	0|0|2	0.76	0.88	0.76	6
SLC25A3	6|2|4	0|1|1	0.74	0.99	0.74	6
DNAJA1	3|12|11	0|2|2	0.72	1	0.72	6.5
RPL7A	12|26|12	4|4|5	0.7	1	0.7	3.85
MAZ	2|7|9	0|0|3	0.68	0.95	0.68	6
RPL27	11|10|9	3|3|4	0.67	0.89	0.67	3
TLN1	11|6|1	0|0|0	0.67	1	0.67	60
PRKDC	11|0|8	0|0|0	0.67	1	0.67	63.33
ABCD3	4|0|7	0|0|0	0.67	1	0.67	36.67
THBS1	9|20|0	1|0|0	0.67	1	0.67	29
POLDIP3	0|4|4	0|0|0	0.67	1	0.67	26.67
IMMT	6|1|4	0|1|0	0.67	1	0.67	11

RPA1	0|3|5	0|0|0	0.66	1	0.66	26.67
NDUFA4	3|1|4	0|0|0	0.66	1	0.66	26.67
ILK	4|3|1	0|0|0	0.66	1	0.66	26.67
NRAS	3|21|0	0|0|0	0.66	1	0.66	80
TECR	3|3|0	0|0|0	0.66	0.99	0.66	20
GALK1	3|9|0	0|0|0	0.66	1	0.66	40
PGRMC1	5|3|1	0|0|0	0.66	1	0.66	30
PRPF19	2|1|4	0|0|0	0.65	1	0.65	23.33
DNAJA3	3|0|2	0|0|0	0.65	0.99	0.65	16.67
C8ORF33	2|3|0	0|0|0	0.65	0.99	0.65	16.67
ATIC	6|2|0	0|0|0	0.65	1	0.65	26.67
RNF138	1|3|2	0|0|0	0.65	0.99	0.65	20
CCT8	6|0|2	0|0|0	0.65	1	0.65	26.67
HSP90B1	3|0|2	0|0|0	0.65	0.99	0.65	16.67
POSTN	3|2|0	0|0|0	0.65	0.99	0.65	16.67
SON	0|2|3	0|0|0	0.65	0.99	0.65	16.67
TUBA1C	27|23|29	0|0|21	0.65	0.81	0.65	3.76
SLC25A1	5|0|2	0|0|0	0.65	1	0.65	23.33
XRCC6	0|3|10	0|1|0	0.65	1	0.65	13
AGK	0|7|2	0|0|0	0.65	1	0.65	30
CCT7	4|0|2	0|0|0	0.65	1	0.65	20
DDX20	0|2|2	0|0|0	0.63	0.95	0.63	13.33
F13A1	2|0|2	0|0|0	0.63	0.95	0.63	13.33
DHCR7	2|1|2	0|0|0	0.63	0.95	0.63	16.67
SNRPB	0|2|2	0|0|0	0.63	0.95	0.63	13.33
EHD3	2|2|0	0|0|0	0.63	0.95	0.63	13.33
APOE	2|2|1	0|0|0	0.63	0.95	0.63	16.67
STAU1	3|11|15	5|3|2	0.63	1	0.63	2.9
RAC1	6|2|6	0|0|3	0.62	0.82	0.62	4.67
SHROOM3	0|6|4	0|1|1	0.62	0.99	0.62	5
ITIH2	4|3|2	2|0|0	0.6	0.78	0.6	4.5
EIF4A1	2|3|4	0|0|2	0.6	0.78	0.6	4.5
SKP1	3|2|4	0|0|2	0.6	0.78	0.6	4.5
RPLP0	11|8|10	2|3|5	0.59	0.89	0.59	2.9
C1QBP	9|1|9	1|0|4	0.59	0.88	0.59	3.8
RPL15	13|14|17	3|4|8	0.58	0.96	0.58	2.93
RPL36AL	7|0|7	2|0|3	0.58	0.87	0.58	2.8
EIF3C	1|3|7	0|1|1	0.55	1	0.55	5.5
GAPDHS	5|3|3	0|0|3	0.54	0.73	0.54	3.67
PCBP1	3|6|6	0|3|2	0.5	0.71	0.5	3

Supplementary Table 4

Significant interactors (SAINT > 0.5) in iPSC FXS-derived neurons treated with FMRP N-tat

Genes	Counts (1-297)	Counts (Not treated)	AvgP	MaxP	SAINT Score	Fold Change
FXR1	28|25|40|24	1|1|0|0	1	1	1	58.5
RPS4Y1	13|13|21|27	0|0|0|0	1	1	1	185
NCL	27|25|34|34	8|6|1|7	1	1	1	5.45
RPL21	9|5|9|14	0|0|0|0	1	1	1	92.5
FXR2	39|40|58|80	0|0|0|0	1	1	1	542.5
RPL7A	10|12|31|16	1|0|2|0	1	1	1	23
RPL3	13|15|30|23	2|3|3|0	1	1	1	10.12
MAP7D1	12|9|8|11	1|1|2|0	1	1	1	10
RPL35A	6|6|7|8	1|0|0|0	1	1	1	27
STAU1	5|3|10|10	0|0|0|0	1	1	1	70
PRKRA	3|3|5|9	0|0|0|0	0.99	1	0.99	50
RPL13	14|11|26|18	3|1|3|3	0.99	1	0.99	6.9
VDAC1	4|5|7|28	0|0|1|0	0.99	1	0.99	44
RPL15	13|15|13|13	5|2|5|1	0.97	1	0.97	4.15
RPL18A	7|5|12|17	1|0|2|0	0.97	1	0.97	13.67
RPL34	4|3|7|7	1|0|0|0	0.96	1	0.96	21
RPL28	14|14|16|11	5|2|4|2	0.95	1	0.95	4.23
RPL8	10|14|22|16	2|2|5|0	0.95	1	0.95	6.89
RPL27	10|11|17|7	2|2|3|0	0.94	1	0.94	6.43
RPL10A	11|10|19|13	4|0|3|0	0.92	1	0.92	7.57
RPL6	14|14|29|29	3|1|7|0	0.9	1	0.9	7.82
RPL7	11|9|22|26	2|1|5|0	0.89	1	0.89	8.5
RPL18	10|7|9|12	0|3|3|0	0.86	0.98	0.86	6.33
RPL10	18|10|25|21	5|4|4|1	0.85	1	0.85	5.29
RPLP2	9|11|13|20	1|3|5|3	0.84	1	0.84	4.42
RPL30	7|5|11|6	3|1|0|0	0.78	0.99	0.78	7.25
RPL13A	4|2|13|14	0|0|2|0	0.76	1	0.76	16.5
RPL4	17|18|41|27	6|1|11|0	0.76	0.99	0.76	5.72
RPL12	5|7|13|17	1|1|4|1	0.75	1	0.75	6
TRIM28	5|5|4|0	1|0|0|0	0.74	0.99	0.74	14
HNRNPU	9|4|13|9	4|0|1|0	0.73	0.96	0.73	7
XPO5	0|3|2|4	0|0|0|0	0.72	1	0.72	22.5
RPS9	14|8|14|21	6|3|3|4	0.72	1	0.72	3.56
VDAC2	4|11|6|24	0|2|2|2	0.71	1	0.71	7.5
RPL14	4|3|10|11	1|1|2|0	0.68	1	0.68	7
RPL5	7|4|12|0	2|0|0|0	0.67	1	0.67	11.5
RPL36	3|3|7|5	1|1|1|0	0.66	0.99	0.66	6
ILF3	4|2|12|0	1|0|0|0	0.64	1	0.64	18
NPM1	3|2|3|4	0|1|0|1	0.59	0.86	0.59	6
SRSF1	1|2|7|10	1|0|1|0	0.57	1	0.57	10
IGF2BP3	2|2|4|0	0|0|1|0	0.53	0.97	0.53	8
DHX30	0|0|4|6	0|0|0|0	0.5	1	0.5	25
C1QBP	0|0|9|12	0|0|0|0	0.5	1	0.5	52.5
XRCC5	5|0|5|0	0|0|1|0	0.5	0.99	0.5	10
INS	0|0|15|20	0|0|0|1	0.5	1	0.5	35
RPLP0P6	9|11|0|0	1|2|0|0	0.5	1	0.5	6.67
HNRNPF	6|0|5|0	0|0|0|0	0.5	1	0.5	27.5
